# Low Vitamin K and Vitamin D Dietary Intake in Patients with Inflammatory Bowel Diseases

**DOI:** 10.3390/nu15071678

**Published:** 2023-03-30

**Authors:** Filippo Vernia, Giorgia Burrelli Scotti, Noemi Sara Bertetti, Giuseppe Donato, Stefano Necozione, Piero Vernia, Nadia Pallotta

**Affiliations:** 1 Division of Gastroenterology, Department of Life, Health and Environmental Sciences, University of L’Aquila, 67100 L’Aquila, Italy; 2 Department of Translational and Precision Medicine, Sapienza University of Rome, 00185 Rome, Italy; 3 Epidemiology Unit, Department of Life, Health and Environmental Sciences, University of L’Aquila, 67100 L’Aquila, Italy

**Keywords:** IBD, vitamin K, vitamin D, diet in IBD

## Abstract

The inadequate dietary intake of Vitamin D and Vitamin K is an easily reversible factor favoring IBD-associated bone loss, but data on Vitamin K are lacking. A 28-item quantitative food frequency questionnaire was administered to 193 IBD patients (89 Crohn’s disease and 104 ulcerative colitis), and 199 controls. Patients’ demographics, clinical and laboratory findings were analyzed in relation to recommended daily allowances. VitD intake was inadequate both in the IBD and control patients (8.3 ± 4.5 µg/day in IBD, 53.1% RDA, and 9.7 ± 5.9 µg/day, 63.2% RDA, respectively). Conversely, the mean ViK intake was less than adequate in IBD, at 116.7 ± 116.3 µg/day (78.7% RDA), and high in controls, at 203.1 ± 166.9 µg/day (138.8% RDA). Nonetheless, due to marked inter-individual differences, diets were severely lacking VitK in 40% of UC and 49% of CD patients, more so in females and those with active disease. The intake of Vit D was non-significantly lower in colitis than that in Crohn’s disease (7.9 vs. 8.7 µg/day). The opposite was observed for VitK (123.5 vs. 107.0 µg/day). Thus, the diet lacks the micronutrients involved in bone wellbeing in a large proportion of IBD patients. While VitD supplementation is the rule, VitK shortages need proactive nutritional intervention.

## 1. Introduction

Patients with inflammatory bowel diseases (IBD) are at an increased risk of osteopenia and osteoporosis. Prevalence approaches 50% in Crohn’s disease (CD) and 30% in ulcerative colitis (UC) [1]. The risk of pathological fractures (86% in CD and 40% in UC) exceeds that of normal controls [2], and impairs the quality of life and prognosis of IBD. This is especially the case in elderly patients.

The pathogenesis of IBD-associated bone loss is multifactorial and still needs to be fully elucidated. Chronic inflammation is of prime importance, due to the unfavorable effect of pro-inflammatory cytokines (IL-1, IL-6, TNFα) on the RANK/RANKL pathway [3]. However, other factors are involved, including long-lasting diseases, the high-dose and protracted use of steroids, surgery, reduced physical activity and deficits of micronutrients, including calcium, vitamin D (VitD) and vitamin K (VitK).

The inadequate intake of calcium and VitD is frequent, and supplements are often prescribed [4]. More recently, attention has been focused on the role of VitK, which is also involved in bone wellbeing in IBD patients [5]. While the relationship between calcium, VitD and bone metabolism is well documented, the clinical relevance of VitK is less clear. Indeed, the inadequate intake of VitK can promote hip fractures [6] and low blood concentrations in IBD, parallel to decreased bone mineral density [7].

In addition to bone metabolism, VitD and VitK shortages affect the modulation of inflammatory responses [8]. Their role in the pathogenesis of IBD and disease activity has been advocated for [9,10], on the basis of epidemiological data.

The requirements of VitD and VitK are largely provided for by the diet. The most relevant dietary source of VitD and calcium is represented by milk and milk derivatives. The same food group, however, contains lactose, which is malabsorbed by a large part of the adult population across the world [11]. The fear that lactose-containing foods induce or worsen abdominal symptoms, bloating and diarrhea, leads to unnecessary restrictions on their intake. This is not rare even in the absence of documented lactose malabsorption, and has been documented in the general population and in patients affected by irritable bowel syndrome [12]. Dietary phylloquinone is primarily provided by green leafy vegetables and to a lesser extent, by some plant oils used in the food industry [13,14]. The metabolic pathways of intestinal bacteria represent an additional source of VitD [15]. While the dietary supply of VitD has been extensively studied in IBD patients, few data are available on VitK [1,16].

The present study was primarily aimed at assessing the dietary intake of VitK in IBD, seeking correlations with demographics and disease characteristics. A high prevalence of their inadequate intake would suggest an additional therapeutic target for the prevention of osteoporosis, and possibly inflammation, in IBD.

## 2. Materials and Methods

**Patients**. The patient population consisted of 193 IBD patients, 100 (51.81%) male and 93 (49.19%) female, who visited our IBD referral center between January 2016 and January 2020. The mean age was 50.69 ± 16.71 years (range 16–84 years). In total, 89 (46.11%) had CD and 104 (53.89%) had UC. The diagnosis of UC/CD was set on the basis of clinical, endoscopic, histological, and cross-sectional imaging findings. In total, 49 UC patients (47.12%) and 51 (57.30%) CD patients were males (Table 1). Patients’ age at diagnosis, localization, the behavior of the disease according to the Montreal Classification [17], current therapy, intestinal surgery, the need of steroids, disease duration, body mass index and smoking status were recorded.

Disease activity was assessed using the Harvey–Bradshaw index (HBI) in CD, and the Partial Mayo score in UC [18,19]. Remission was defined as HBI ≤ 5 for CD and as a partial Mayo score ≤ 1 for UC patients. The C-reactive protein (CRP) and erythrocyte sedimentation rate (ESR) were measured.

**Food intake**. Information on the diet during the week prior to observation was acquired by a dietitian by completing a food frequency checklist. The questionnaire was adapted from a 22-item quantitative FFQ, previously validated for calcium [20] and VitD intake, and integrated with 6 specific questions; these were focused on foods with the highest phylloquinone concentration (100–400 µg VitK/100 g). Green leafy vegetables, including spinach, iceberg lettuce, chicory, beets, turnip tops and rocket salad, as well as eggs, were the main contributors to VitK intake. In accordance with previous studies, using validated FFQ, the food list included country-specific items [21,22] in order to minimize the risk of over and underestimating VitD and VitK intake.

The usual serving size of foods was evaluated in each subject using a photographic atlas of food portions [23]. The dietician assessed and recorded how often each item was consumed. The daily phylloquinone and VitD intake from the diet was calculated by multiplying the frequency and serving size for each portion by the nutrient content of the food.

As the available reference data for vitamin content show marked differences, in the present study, we used the mean value from three different sources (EFSA, United States Department of Agriculture and Istituto Nazionale di Ricerca per gli Alimenti e la Nutrizione). The food composition values we used are shown in Appendix A.

Dietary data from IBD patients were compared with those of the healthy family members accompanying the non-gastroenterological outpatients of our department, and staff members (199 controls).

**Recommended daily allowances and average daily requirements**. Data recorded from patients and controls were expressed as a percent of the recommended daily allowances (RDA) for VitD, using the reference values of 15–20 µg/d [24]. An inadequate intake of VitD was defined as <66% of RDA.

Conversely, the Average Daily Requirements (AR) and Population Reference Intakes (PRI) of VitK differ according to country and eating habits [25,26]. Thus, we chose, as reference AR values, 140 µg/d for females and males up to 59 years old, and 170 µg/d for patients >60 years, as proposed by the Società Italiana di Nutrizione Umana (SINU) [26]. These values are higher than those proposed by NIH and EFSA [24,25], and reflect the Mediterranean diet in our country. VitK data were thus expressed as a percent of the reference intake levels. An inadequate intake of VitK was defined as <66% of AR.

The study was conducted according to the guidelines laid down in the Declaration of Helsinki and did not include procedures that are not part of usual outpatient visits, including the assessment of patients’ diet and nutritional status.

### Statistical Analysis

The SAS statistical package (version 9.4, 2002–2012 by SAS Institute Inc., Cary, NC, USA) was used for statistical analysis. Data were compared using the T-Test for normally distributed parameters, the Mann–Whitney Test was used for skewed data and the chi-square test was used for proportional data. *p* < 0.05 was considered statistically significant.

The sample size by far exceeded the figures required to grant an 80% statistical power, with a 0.05 alpha error. For Vit K, 40 subjects were needed in the patient and control group; a 50% difference between the patients and controls was hypothesized, with 100% SD of the mean observed value. For Vit D, 63 subjects were needed in the patient and control groups; a 20% difference between the patients and controls was hypothesized, with 50% SD of the mean observed value.

## 3. Results

### 3.1. VitK and VitD Intake in Inflammatory Bowel Disease Versus Controls

The mean VitK intake was 117.86 ± 126.72 SD µg/day in the IBD group, corresponding to 78.72% of the RDA, and 203.12 ± 166.93 SD µg/day in the controls (138.79% RDA). The difference between the healthy controls and IBD patients was statistically significant (*p* < 0.001) (Table 2).

The mean VitD intake was 8.23 ± 4.57 SD µg/day in the IBD group (53.76% RDA) and 9.67 ± 5.91 µg/day (63.58% RDA) in the controls (*p* = 0.03) (Table 2).

Within the IBD group, the total daily VitK intake was non-significantly lower in CD (110.64 ± 120.11 µg/day, 75.55% of RDA) than in UC (124.04 ± 132.38 SD µg/day, 81.43% of RDA).

Although the mean values exceeded the RDA in all the study groups, the intake of VitK showed wide individual variations. Thus, 53.73% of IBD patients (50.96% CU and 61.80% CD) had a markedly reduced VitK intake. The same proved true for 30% of the controls.

Intake data, the percent of RDA and the number of patients with an inadequate (<66%) intake of Vit D and K are reported in Table 3.

### 3.2. VitK and VitD Intake in Relation to Gender

The mean daily VitK and VitD intake in the IBD patients and controls in relation to gender are reported in Table 3.

The daily intake of VitD in the control males and females was 11.36 ± 6.68 µg/day and 8.78 ± 5.26 µg/day (74.96% and 57.59% of RDA), respectively (*p* = 0.007). VitK intake in the control males was 183.44 ± 143.39 µg/day and 212.96 ± 177.81 µg/day in females (*p* = n.s.). The percent of RDA and the proportion of patients with an intake below 66% of RDA are reported in Table 3.

The VitK intake was 103.20 ± 107.38 µg/day and 131.49 ± 141.56 µg/day in females and males in the IBD group, respectively. Figures slightly exceeded the RDA (70.85% and 86.04%, respectively).

Vitamin D intake was of the same order in female and male patients (8.04 ± 4.57 µg/day, and 8.40 ± 4.58 µg/day, respectively). The percent of RDA and the proportion of patients with an intake lower than 66% of RDA are reported in Table 3.

The difference between the IBD group and controls was significant in both genders for VitK (*p* ≤ 0.001); in males, this was only (*p* = 0.004) as far as VitD was concerned. The intake of VitD in females did not differ between the IBD cases and controls (*p* = 0.38).

### 3.3. VitK and VitD Intake in Different Age Groups

The recorded intake of VitK and VitD in the IBD group according to age is reported in Table 2. The mean daily intake of VitK was low in patients aged < 40 and >60 (70.08 and 67.94%, respectively) and normal in those aged 41–60 (92.65%).

Controls showed adequate mean values for VitK assumption in all age groups (<40 138.01% RDA; 41–60 166.07%; >61 100.74%). These values significantly exceeded those of the IBD patients in the three cohorts (*p* < 0.0001, *p* < 0.0001 and *p* = 0.05, respectively).

The participants’ VitD mean daily intake was below the RDA in all age groups, both in the control and IBD group. Despite overall lower figures in the IBD group in the three cohorts, the significance level was not attained (Table 3).

### 3.4. Site, Extent of Lesions and Disease Activity in IBD Patients

Disease activity did not affect the mean daily VitK intake, which was adequate in activity and remission, despite non-significantly lower values in the active disease group (108.93 ± 105.37 µg/day vs. 122.58 ± 135.15 µg/day, in remission; *p* = 0.50) (Table 3).

Conversely, VitD intake was significantly reduced in patients with active disease (7.17 ± 4.05 µg/day versus 8.72 ± 4.7 µg/day, in remission, *p* = 0.01).

The participants’ body mass index (<20, 20–25, >26 kg/m^2^) did not correlate with the intake of VitK and VitD.

Disease localization in CD markedly influenced the daily intake (Table 4). Although VitK was below the RDA in all subgroups, ileocolonic disease was associated with lower values (97.82 ± 119.14; 67.99%), compared to small bowel (124.36 ± 125.34, 82.95%) or colonic disease (115.99 ± 117.08; 79.82%). The difference between the three groups was non-significant (*p* = 0.36). The same was true for VitD intake, (L1 7.85 ± 4.63, 51.49%; L2 9.53 ± 4.19, 62.38%; L3 9.34 ± 4.66, 61.10%) (*p* = 0.17).

The disease behavior in CD influenced to a greater extent VitK than VitD ingestion. The VitK daily consumption approached the reference values in patients with an inflammatory pattern, as expected, but not in those with structuring or perforating CD. The relatively small number of patients in each group prevented the values from reaching the significance level.

VitD intake was reduced in the three groups of patients (Table 4).

Disease extension influenced VitK, and to a lesser extent, VitD intake in UC (Table 5). Like in CD, the sample size prevented the values from reaching the significancy threshold for VitK.

Previous surgery did not affect the dietary habits as far as VitD and Vit K are concerned (Table 6 and Table 7).

## 4. Discussion

Recording the dietary intake of micronutrients in IBD offers valuable information on the individual risks of experiencing clinically relevant deficits. While VitD status is effectively estimated by dosing 25-hydroxy Vitamin D3, the issue is more complex for VitK. Direct serum phylloquinone measurements do not offer a reliable evaluation of VitK levels, due to its short half-life [24]. Blood concentrations are influenced by the daily intake more than by the total body storage. Indirect evaluations, based on VitK-dependent enzymes, such as uncarboxylated osteocalcin or PIVka, are thus preferred, but are not widely available. In bedside clinical practice, isolated abnormal prothrombin time (PT) is considered to be suggestive of VitK deficiency and thus prompts supplementation. However, PT does not represent an effective surrogate biomarker of VitK deficiency, as only the proportion of VitK that is not used by liver biosynthesis is available for bone metabolism [1]. Thus, VitK deficiency impairs bone metabolism before any effect on coagulation is observed; indeed, bone is susceptible to less marked VitK shortage than liver function.

The importance of VitD for bone metabolism has been known for decades. VitD, however, also has well documented anti-inflammatory effects, and modulates innate and adaptive immune responses [27,28,29,30]. The expression of the tight junction constituent claudin-2 is stimulated by VitD, directly affecting mucosal function. Thus, VitD shortage increases gut permeability and favors the migration of antigenic molecules and bacteria through the mucosal barrier. Low concentrations of 1,25(OH)2D in the blood in IBD patients correlate with morbidity, the risk of corticosteroid therapy, hospitalization, disease severity and a worse quality of life [31,32]. Nonetheless, the administration of VitD does not improve CRP levels in patients with CD and UC [33].

The pathophysiological implications of VitK deficiency in IBD are less clear. In addition to its anti-hemorrhagic role, VitK exerts favorable effects on inflammation, atherogenesis, oxidative processes and cancer prevention through the carboxylation of Gla-rich proteins [34,35,36]. Its deficit represents an additional mechanism that favors osteoporosis [37,38,39]. Carboxylated Gla residues are indeed essential for bone matrix metabolism and the biological activity of Glaprotein, S protein and the osteocalcin bone modulator [40]. Increasing evidence 250 supports the role of VitK on intestinal flora and gut permeability [41], as well as the favorable effects of VitK supplementation [42]. Indeed, VitK deficiency leads to the exacerbation of inflammation and enhances histological damage in animal models [43]. The alterations are partially reversed by VitK1 supplements.

In our study, we demonstrated that IBD patients have a significantly lower intake of VitK and VitD compared to controls, more so in active disease cases.

The widely shared opinion that milk and lactose-containing foods worsen diarrhea is a prime determinant of inadequate calcium and VitD intake in IBD [44,45] patients; this is even the case in the absence of documented lactose malbsorption and intolerance, and is irrespective of the lactose content of individual servings. The unfavorable metabolic effects of low VitD intake are further amplified by the inadequate sunlight exposure documented in IBD patients [46]. This is clinically relevant, as sunlight exposure affects the conversion of VitD precursors to its activated form and is more important than diet in determining plasma concentrations of 25(OH)D.

In addition to the restrictions imposed on lactose-containing food, IBD patients avoid vegetables in the fear of worsening diarrhea. Minimizing the intake of insoluble fiber is advisable in stricturing or perforating CD, but restriction is unnecessary in inflammatory CD and UC. This attitude is not rare and has profound metabolic implications. Green leafy plants, which are well represented in the Mediterranean diet, are, indeed, the primary dietary source of phylloquinone (VitK1), providing over 90% of requirements [47]. Besides directly favoring VitK shortage, a marked reduction in vegetables and fermentable carbohydrates in the diet decreases the intraluminal production of other useful compounds, such as short-chain fatty acids. This may worsen, rather than improve, diarrhea. Butyrate indeed represents the primary energy substrate for colonocytes, and enhances the absorption of sodium and water, reducing the colonic output. It also improves mucosal permeability, modulates the expression of anti-inflammatory and mucus-controlling genes, and proves clinically effective in UC patients [48,49,50].

The role of dietary intervention therapies in IBD, consisting of the reduction or exclusion of food that could worsen IBD, has recently been reviewed [51]. They include refined carbohydrate diets, low-processed red meat diets, the so-called Alberta-based anti-inflammatory diet, the carrageenan-free diet and the milk-free diet. The review could not reach firm conclusions on the possible benefits, or harms, of these restriction diets. Conversely, active supportive intervention is promising, being focused on a reduction in oxidative stress and proinflammatory cytokines [52]. The modulation of colonic intraluminal content may also prove useful in IBD, resulting in changes in the gut microbiome and bacterial metabolic byproducts, vitamins included [53].

The present data confirm that VitD intake was abnormally low in IBD patients and in controls [1,11,54]. Conversely, the mean VitK intake in this series was only slightly below the RDA. This finding is unexpected [55]. However, focusing one’s attention on the mean values may be misleading when the interindividual variation is wide. The diet of some patients reflected that of the background population and included numerous servings of vegetables, providing more than adequate dietary phylloquinone. Conversely, the intake of VitK was below 66% of RDA in 40% of UC patients and about half of CD patients. Thus, despite normal PT values in over 90% of cases (data not reported), a shortage of VitK was likely present in a considerable proportion of IBD patients. The dosage of VitK-dependent enzymes in IBD patients being reviewed in order to confirm the hypothesis.

## 5. Conclusions

The present study confirms that the diet of IBD patients often lacks VitD and VitK. The restriction of milk and dairy products results in an inadequate intake of calcium and vitamin D. A modified diet that is low in insoluble fibers is often suggested to or self-prescribed by IBD patients, irrespective of the presence or absence of intestinal strictures, which prompt restrictions. Almost half of our patients limited the consumption of vegetables, green leafy vegetables included. This led to an inadequate supply of phylloquinone, and possibly to a shortage of VitK even in the absence of altered prothrombin time.

VitD supplements are part of the therapeutic strategy targeted at IBD patients. Iron deficit is actively sought after and treated with specific replacement regimens [56]. Vitamin K supplementation is advised in bone and inflammatory rheumatic disease, chronic renal failure and for the prevention of vascular calcification and cardiovascular disease [57,58,59,60]; however, is it hardly mentioned in the nutritional guidelines for IBD patients. This attitude should change in order to reduce the adjunctive risk factor of osteoporosis in already high-risk patients, prevent coagulation defects and possibly help modulate inflammatory responses in IBD.

## Figures and Tables

**Table 1:** Patient Characteristics.

Male	100 (51.81%)
Female	93 (48.19%)
UC	104 (53.89%)
E1	25 (24.04%)
E2	37 (35.58%)
E3	42 (40.38%)
CD	89 (46.11%)
L1	33 (37.07%)
L2	10 (11.24%)
L3	46 (51.69%)
Inflammatory	51 (57.30%)
Stricturing	26 (29.21%)
Perforating	12 (13.49%)
IBD Activity	59 (30.57%)
IBD Remission	134 (69.43%)
Age	
<40	57 (29.53%)
41–60	82 (42.49%)
>60	54 (27.98%)
Previous surgery	30 (15.54%)
No Surgery	163 (84.46%)

UC—Ulcerative Colitis, CD—Crohn’s Disease, IBD—Inflammatory Bowel Diseases. Data are expressed in absolute numbers. Percent of patients within brackets. Patient population and demographics, disease activity and previous surgery.

**Table 2:** Vitamin K and D intake in IBD and controls in relation to gender and age.

**Vitamin K Intake** **(μg/day)**	**IBD**	**SD**	**Controls**	**SD**	***p* Value**
**Total**	117.86	126.72	203.12	166.93	*p* < 0.001
**Males**	131.49	141.56	183.44	143.39	*p* < 0.001
**Females**	103.20	107.38	212.96	178.81	*p* < 0.01
**Age Groups**					
**<40**	102.72	113.50	193.22	137.84	*p* < 0.0001
**41–60**	131.15	115.96	239.27	202.28	*p* = 0.05
**>60**	115.50	153.24	171.26	160.02	*p* < 0.0001
**Vitamin D Intake** **(μg/day)**	**IBD**	**SD**	**Controls**	**SD**	***p* Value**
**Total**	8.23	4.57	9.67	5.91	*p* = 0.03
**Males**	8.40	4.58	11.36	6.68	*p* = 0.004
**Females**	8.04	4.57	8.78	5.26	n.s. (*p* = 0.38)
**Age Groups**					
**<40**	8.71	5.03	10.53	6.70	n.s. (*p* = 0.14)
**41–60**	8.73	4.58	9.53	5.59	n.s. (*p* = 0.56)
**>60**	7.06	3.84	8.06	4.04	n.s. (*p* = 0.24)

IBD: Inflammatory Bowel Diseases. n.s.: non-significant. Data expressed in μg/day.

**Table 3:** Vitamin K and Vitamin D intake in IBD patients in relation to gender, age, disease activity and diagnosis.

Nr of Pts	Vitamin K * (% RDA)	SD	*p* Value	Percent Pts with <66% RDA	Vitamin D * (% RDA)	SD	*p* Value	Percent Pts with <66% RDA
**Overall** **IBD series** **193**	117.86 (78.72)	126.72		55.73%	8.23 (53.76)	4.57		71.35%
**Males** **100**	131.49 (86.04)	141.56	n.s.	55.0%	8.40 (54.77)	4.58	n.s.	71.0%
**Females** **93**	103.20 (70.85)	107.38		56.99%	8.04 (52.66)	4.57		72.04%
**Age** **<40 (a)** **57**	102.72 (70.08)	113.50	*p*_a–b_ = n.s. *p*_b–c_ = n.s. *p*_a–c_ = n.s.	58.93%	8.71 (58.04)	5.03	*p*_a–b_ = n.s. *p*_b–c_ = n.s. *p*_a–c_ = n.s.	64.29%
**41–60 (b)** **82**	131.15 (92.65)	115.96		50.0%	8.73 (58.18)	4.58		68.29%
**>60 (c)** **54**	115.60 (67.94)	153.24		61.11%	7.06 (43.22)	3.84		83.33%
**Activity** **59**	108.93 (71.73)	105.37	n.s. (*p* = 0.05)	53.45%	7.17 (47.02)	4.05	*p* = 0.01	84.48%
**Remission** **134**	122.58 (82.32)	135.15		56.72%	8.72 (56.92)	4.70		65.67%
**UC** **104**	124.04 (81.43)	132.38	n.s.	50.96%	7.71 (50.27)	4.50	n.s.	77.88%
**CD** **89**	110.64 (75.55)	120.11		61.80%	8.83 (57.84)	4.59		64.04%

IBD: Inflammatory Bowel Diseases, RDA: recommended daily allowances, n.s.: non-significant. Data expressed in absolute values (μg/day *). Percent of RDA: between brackets. Column 5 and 9: percent of patients with a daily intake below 66% of RDA.

**Table 4:** Vitamin K and D intake, in Crohn’s Disease, in relation to disease location and behavior.

CD Nr of Pts	Vit K μg/day	SD	*p* Value	% RDA	Vit D μg/day	SD	*p* Value	% RDA
**L1** **33**	124.35	125.34	*p*_l1–l2_ = n.s. *p*_l1–l3_ = n.s. *p*_l2–l3_ = n.s.	82.95	7.85	4.63	*p*_l1–l2_ = n.s. *p*_l1–l3_ = n.s. *p*_l2–l3_ = n.s.	51.49
**L2** **10**	115.99	117.08		79.82	9.53	4.19		62.38
**L3** **46**	97.82	119.14		67.99	9.34	4.66		61.10
**inflammatory (a)** **51**	129.26	132.17	*p*_a–b_ = n.s. *p*_b–c_ = n.s. *p*_a–c_ = n.s.	89.29	9.43	4.78	*p*_a–b_ = n.s. *p*_b–c_ = n.s. *p*_a–c_ = n.s.	62.00
**structuring (b)** **26**	97.71	106.35		64.67	8.74	4.29		56.53
**perforating (c)** **12**	61.80	68.52		42.11	6.83	3.62		45.40

CD: Crohn’s Disease. Disease localization: L1 terminal ileum, L2 colon, L3 ileo-colon. n.s.: non-significant. Data expressed in μg/day and percent of RDA.

**Table 5:** Vitamin K and D intake, in Ulcerative Colitis, in relation to disease extent.

Uc Nr Of Pts	Vit K μg/day	SD	*p* Value	% RDA	Vit D μg/day	SD	*p* Value	% RDA
**E1** **25**	185.64	203.43	*p*_E1–E2_ = n.s. *p*_E2–E3_ = n.s. *p*_E1–E3_ = n.s.	123.31	8.39	5.51	*p*_E1–E2_ = n.s. *p*_E2–E3_ = n.s. *p*_E1–E3_ = n.s.	53.10
**E2** **37**	106.44	104.74		72.26	7.89	4.14		52.12
**E3** **42**	108.07	90.67		67.85	7.26	4.28		47.57

Disease extent: E1 proctitis, E2 left-sided colitis, E3 extensive colitis. n.s.: non-significant. Data expressed in μg/day and percent of RDA.

**Table 6:** Vitamin K and D intake and surgery in CD.

IBD Nr of Pts	Vit K μg/day	SD	*p* Value	% RDA	Vit D μg/day	ASD	*p* Value	% RDA
**Surgery**			n.s.				n.s.	
**25**	111.31	137.84		74.45	7.67	4.60		50.54
**No surgery**								
**64**	109.23	114.55		75.09	9.26	4.58		60.53

CD: Crohn’s disease. n.s.: non-significant. Data expressed in μg/day.

**Table 7:** Vitamin K and D intake and previous surgery in UC.

IBD Nr of Pts	Vit K μg/day	SD	*p* Value	% RDA	Vit D μg/day	ASD	*p* Value	% RDA
**Surgery**			n.s.				n.s.	
**5**	203.73	73.65		131.69	7.73	4.80		51.52
**No surgery**								
**99**	120.83	135.08		79.24	7.82	4.54		50.88

UC: Ulcerative Colitis. n.s.: non-significant. Data expressed in μg/day.

## Appendix A

**Table A1:** Vitamin K and Vitamin D food composition values, data expressed in µg/100 g.

	Vitamin K	Vitamin D
Milk	0.25	1.6
Yoghurt	0.2	0.05
Hard Cheese	1.95	0.25
Parmesan	0	0.5
Semi-Hard Cheese	2.4	0.25
Fresh Cheese	1.7	0.25
Pasta	0.1	0
Rice	0.1	0
Bread	1.2	0
Potatoes	1.9	0
Fish	0.2	13
Meat	1.46	1
Egg	0.3	2
Legumes	7.8	0
Vegetables	8	0
Fresh Fruit	1.8	0
Ice cream	0.86	0.2
Milk/White Chocolate	1.6	0
Chard	163.65	0
Spinach	246.8	0
Red Chicory	127.6	0
Lettuce	66.5	0
Rocket Salad	54.3	0
Turnip Greens	125.5	0
